# Effects of Probiotic *Saccharomyces boulardii* Supernatant on Viability, Nano-Mechanical Properties of Cytoplasmic Membrane and Pro-Inflammatory Gene Expression in Human Gastric Cancer AGS Cells

**DOI:** 10.3390/ijms24097945

**Published:** 2023-04-27

**Authors:** Babak Pakbin, Samaneh Allahyari, Shaghayegh Pishkhan Dibazar, Leila Zolghadr, Neda Karami Chermahini, Wolfram Manuel Brück, Thomas B. Brück, Razzagh Mahmoudi

**Affiliations:** 1 Institute for Life Technologies, University of Applied Sciences Western Switzerland Valais-Wallis, 1950 Sion, Switzerland; b.pakbin@tum.de (B.P.);; 2 Werner-Siemens Chair of Synthetic Biotechnology, Department of Chemistry, Technical University of Munich (TUM), Lichtenberg Str. 4, 85748 Garching bei München, Germany; samaneh.alahyari66@gmail.com; 3 Medical Microbiology Research Center, Qazvin University of Medical Sciences, Qazvin 34197-59811, Iran; 4 Department of Immunology, Faculty of Medical Sciences, Tarbiat Modares University, Tehran 14119-44961, Iran; 5 Department of Chemistry, Imam Khomeini International University, Qazvin 34149-16818, Iran; 6 Department of Medicine Biotechnology, Faculty of Allied Medicine, Qazvin University of Medical Science, Qazvin 34197-59811, Iran

**Keywords:** *Saccharomyces boulardii*, postbiotic, anticancer properties, AGS cell line

## Abstract

Background: Gastric cancer has been recognized as the second most probable cause of death in humans from cancer diseases around the world. Postbiotics, supernatant, and metabolites from probiotic microorganisms have recently been used widely to prevent and treat cancer diseases in humans, without any undesirable side effects. This study explores the antiproliferative and antitumor activities of the probiotic *Saccharomyces cerevisiae* var. *boulardii* supernatant (SBS) against AGS cancer cells, a human gastric adenocarcinoma cell line. Methods: We evaluated cell growth inhibitory and mechanical properties of the cytoplasmic membrane and the downregulation of *survivin* and proinflammatory genes in AGS cells treated with SBS after 24 and 48 h. Results: SBS significantly inhibits the AGS cell growth, and the concentrations with IC_50_ values after 24 and 48 h treatments are measured as 2266 and 1956 µg/mL, respectively. Regarding the AFM images and Young`s modulus analysis, SBS significantly induces morphological changes in the cytoplasmic membrane of the treated AGS cells. Expression of *survivin*, *NFƙB*, and *IL-8* genes is significantly suppressed in AGS cells treated with SBS. Conclusions: Considering the antitumor activities of SBS on AGS cell line, it can be regarded as a prospective therapeutic and preventive strategy against human stomach cancer disease.

## 1. Introduction

Gastric cancer (GC) is known as one of the leading causes of death due to cancer and is the fifth most common cancer around the world. This chronic disease has recently been regarded as a major public health issue and a significant source of mortality, mostly in developed countries [1]. More than 950 000 new cases of GC are reported annually and it is also estimated that more than 720,000 patients die due to this cancer each year around the world. GC is also recognized as the third main contributor to the global burden of disability-adjusted life-years caused by cancer diseases, following lung and liver cancers [2]. Low-fiber diets, high salt intake, age, genetic factors, and Helicobacter pylori infection are the main known risk factors causing GC in humans [3]. The incidence rate of gastric adenocarcinoma is increasing sharply in both developed and developing countries. Various preventive and therapeutic strategies have been suggested and are being attempted against GC in humans. Surgery and chemotherapy remain the most effective and curative therapeutic strategies to treat patients with GC [3,4]. Regarding the cognitive and brutal side effects of these strategies in beating GC, several novel preventive and therapeutic alternatives based on natural biological products, such as plant extracts, microbial metabolites, and biomass supernatant, have recently been evaluated and presented. These health-promoting materials show strong antitumor activities and decrease the viability of different human cancer cells [4,5,6].

Probiotics are defined as live microorganisms that induce health benefits in human or animal hosts through specific activities in the gastrointestinal tract while being digested in adequate amounts [7]. The microorganisms with probiotic activity most commonly used in the food and pharma industries are the bacteria of the genera *Lactobacillus*, *Bifidobacterium*, and *Streptococcus*, and fungal probiotics including *Saccharomyces cerevisiae* var. *boulardii* and *Kluyveromyces marxianus* [8,9]. Probiotic organisms confer diverse health benefits, including cell-mediated immunity stimulation, epithelial cell integrity, carcinogenic compound detoxification, lactose intolerance alleviation, serum cholesterol reduction, competition for adhesion and nutrients with pathogens, immune globulin A production, and the secretion of different active metabolites such as bacteriocins and organic acids [10,11]. In spite of various health-promoting actions, a significant number of studies undermined the safety and effectiveness of probiotics, especially in vulnerable and high-risk people. Therefore, the interest in the safe surrogate groups of probiotics, including microbial metabolites, cell-free extracts, and supernatants of probiotic biomass, has been growing [12,13,14]. *S. boulardii* is known as a therapeutic probiotic yeast utilized for the prevention and treatment of acute diarrhea in infants and young children, as well as for the treatment for chronic gastrointestinal disorders such as inflammatory bowel disease [15]. Considering the opportunistic nature of this probiotic yeast, some reports pointed out the fungemia caused by *S. boulardii* in immunocompromised and vulnerable patients [16]. Therefore, postbiotic products of *S. boulardii* including supernatant, cell-free extract, and metabolites are highly recommended to be used to prevent and treat cancer diseases in humans [17,18]. Previously, we evaluated the anticancer potential of *S. boulardii* metabolites and supernatant against different human cancer cell lines, including MCF7 (human breast cancer), caco-2 (human colon cancer), and EPG85-257P (human stomach cancer) cell lines [5,6,17,18]. We were motivated to evaluate the antitumor properties of SBS against AGS cancer cells, a human gastric adenocarcinoma cell line.

## 2. Results

**Figure 1:**
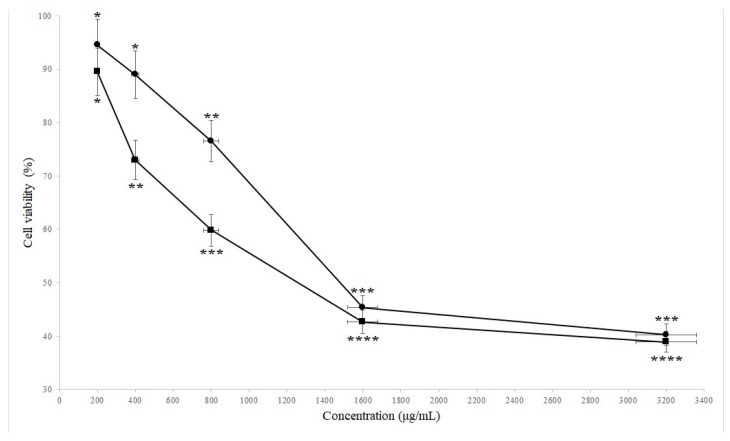
Cell viability of AGS cells treated with different concentrations of SBS, including 200, 400, 800, 1600, and 3200 µg lyophilized dried SBS in 1 mL RPMI cell culture media, evaluated by the MTT assay in comparison with the negative control (treated with DMSO without any significant anti-proliferative effects against AGS cell line). Filled circles and squares represent treatments after 24 and 48 h, respectively. *, **, *** and **** indicate significant differences (*p* < 0.05).

We demonstrated the cytotoxic and antitumor activities of SBS against human stomach cancer cells in this study. Cell viability of AGS cells treated with different concentrations of SBS after 24 and 48 h are shown in Figure 1. SBS significantly (*p* < 0.05) reduces the viability of AGS cells after 24 and 28 h in comparison with the negative controls (cells treated with DMSO in the same volume). We also observe that AGS cell viability decreases dose-dependently. The significantly (*p* = 0.012) highest antitumor activity of SBS against AGS cells after 24 and 48 h is observed with the 1600 µg/mL concentration. However, significantly (*p* = 0.032) higher cytotoxic activity against stomach cancer cells is observed after 48 h than after 24 h treatment with SBS. After observation of the anticancer properties of SBS against AGS cells, we were encouraged to investigate the nano-mechanical and morphological properties and the expression of *survivin* and proinflammatory genes in human stomach cancer cells treated with SBS. The concentration of SBS needed to reduce the AGS cells by 50% (the half maximal inhibitory concentration, IC_50_) was considered after 24 and 48 h to treat the cells and analyze the gene expression and mechanical properties. SBS concentrations with IC_50_ values for 24 and 48 h treatments are calculated at 2266 and 1956 µg/mL, respectively.

**Figure 2:**
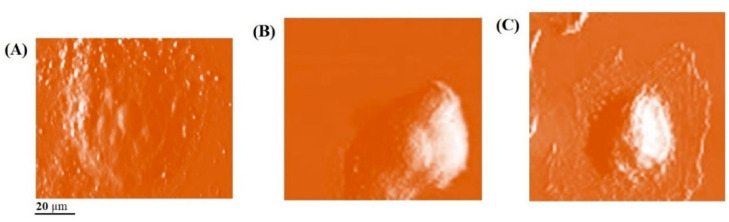
AFM images of AGS cells treated with DMSO (**A**) and SBS after 24 (**B**) and 48 h (**C**). Areas with white color indicate cytoplasmic membrane changes.

Morphological and nano-mechanical features of the cytoplasmic membrane of treated AGS cells were assessed by using the AFM method in this study. The AFM images and Young`s modulus analysis are shown and described in Figure 2 and Table 1, respectively. Significantly (*p* < 0.05) lower levels of cell flexibility and higher levels of Young`s modulus and elastic modulus are observed in AGS cells treated with SBS compared to the control sample (treated with DMSO). However, there are no significant (*p* < 0.05) differences in mechanical properties between the treatments after 24 and 48 h. White areas on AGS cells in AFM images indicate morphological changes, induced apoptosis, and cytoplasmic membrane pores. Compared with the control cells (treated with DMSO), more white areas are observed in the AFM images of AGS cells treated with SBS after 24 and 48 h. Regarding the results of the AFM analysis, we observe that SBS significantly leads to more changes in the morphological and mechanical properties of the AGS cytoplasmic membrane.

**Figure 3:**
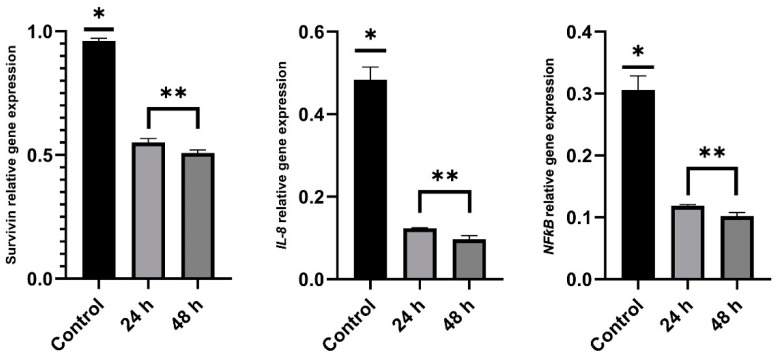
Relative expression of *survivin*, *IL-8*, and *NFƙB* genes in AGS cells treated with DMSO and SBS after 24 and 48 h. * and ** indicate significant differences (*p* < 0.05).

Survivin is an intracellular protein encoded by the *survivin* gene that belongs to the apoptosis inhibitor family gene, and is highly expressed in the most common human tumor cells. This protein plays as important role in regulating cell proliferation of AGS cells. Higher expression of this gene correlates with more viability of gastric cancer cells, poor clinical outcome, and more aggressive disease [5,18]. Anticancer drugs and compounds decrease the expression levels of the *survivin* gene in cancer cells. Pro-inflammatory genes such as *NFƙB* and *IL-8* are over-expressed in cancer cells. Antitumor agents also induce suppression of pro-inflammatory genes in treated cancer cells. In this study, we evaluated the expression levels of *survivin*, *NFƙB*, and *IL-8* genes in AGS cells treated with SBS in concentrations with IC_50_ values by using real-time PCR and 2^−ΔΔCt^ assays. Figure 3 shows the relative expression of the *survivin* gene in stomach cancer cells treated with SBS after 24 and 48 h. The expression of the *survivin* gene is significantly (*p* < 0.05) suppressed in AGS cells treated with SBS. Relative expression of *IL-8* and *NFƙB* genes are also shown in Figure 3, respectively. As can be seen in these figures, SBS treatment significantly (*p* < 0.05) reduces the expression levels of *IL-8* and *NFƙB* genes in AGS cells. However, there are no significant differences (*p* < 0.05) in the expression of *survivin*, *IL-8*, and *NFƙB* genes in AGS cells between the 24 and 48 h SBS treatments. In general, we find that SBS induces downregulation of *survivin*, *IL-8*, and *NFƙB* genes in human stomach cancer cells.

## 3. Discussion

In 2020, stomach cancer was recognized as the fifth most malignant cancer disease and the fourth leading cause of death from cancer in the world, with approximately more than one million new cases and 800 000 deaths annually [19]. Stomach cancer incidence correlates with increasing age, and it has been chiefly diagnosed in men and in Asian and South American countries [20]. In recent years, different nature-based preventive and therapeutic strategies against stomach cancer disease in humans have been developed and evaluated [21]. Probiotics and postbiotic compounds have also widely been used as antitumor and cancer-preventive agents [22]. Previously, we evaluated anticancer and apoptosis-inducing properties of supernatant and metabolites of probiotic *S. boulardii* against different human cancer cells such as colon (caco-2), daunorubicin-resistant stomach (EPG85-257RDB), and breast (MCF-7) cancer cell lines [5,6,17,18]. AGS cell line is commonly used as a human stomach cancer cell line in in vitro study models to evaluate the anticancer properties of different compounds and therapeutic strategies against human gastric cancers [23]. In this study, we assessed the anticancer potential of SBS against human stomach cancer cells in an in vitro model (AGS cell line). 

We find that SBS exerts cytotoxic and antiproliferative effects on human stomach cancer cells. Several studies also investigated and reported the antiproliferative activity of natural compounds from plant and microbial strains against human gastric adenocarcinoma cells in an AGS cell line study model. Wu et al. (2008) investigated the antiproliferative effects of luteolin, a flavonoid compound extracted from plant kingdoms with a wide range of health-promoting activities, against AGS cell lines. They found that luteolin inhibited AGS cell growth in time and dose-dependent manners [24]. Pan et al. (2013) reported the cancer cell growth inhibitory activity of lactoferricin against the AGS cell line. As previously mentioned, researchers have recently become interested in the antiproliferative properties of compounds extracted from different microorganisms [25]. Saber et al. (2017) evaluated the anticancer properties of secretions from the probiotic *Kluyveromyces marxianus*, and they showed significant cytotoxic effects of these secretions against the AGS cell line and other human cancer cells [26]. Hwang et al. (2022) also focused on the antitumor properties of heat-killed *Lactobacillus brevis* against AGS cancer cells, and they confirmed that heat-killed suspension of this organism induced an antiproliferative effect on human cancer cells [23]. Moreover, probiotic microbial supernatant and metabolites showed antiproliferative activity on other human cancer cells, such as the supernatant of probiotic lactic acid bacteria and *K. marxianus* cell wall extract against human colon cancer cells [26,27,28]. *S. boulardii* biomass and supernatant are composed of some specific biologically active compounds, such as complex profiles of beta-glucans, commonly leading to antioxidant, antitumor, and immunomodulatory properties [5,6,28,29].

Regarding the growth-inhibitory activity of SBS against the AGS cell line, we explored the morphological properties and expression of *survivin* and pro-inflammatory genes in AGS cells treated with SBS in this study. We find that SBS induces the suppression of pro-inflammatory and *survivin* genes and cytoplasmic membrane morphological changes in treated AGS cell lines. *Survivin* is a protein that inhibits apoptosis, preserves cell viability, and is implicated in mitosis regulation [30]. Downregulation of *survivin* genes and dissociation of the cytoplasmic membrane induce apoptosis and cell death in treated cancer cells [31]. Pro-inflammatory genes such as *IL-8* and *NFƙB* are highly expressed in cancer cells. Antitumor compounds and strategies induce the downregulation of pro-inflammatory genes in cancer cells [5,32,33]. In this study, SBS also suppresses the expression of *IL-8* and *NFƙB* genes in treated AGS cell lines. Previously, we observed that the metabolites of *S. boulardii* led to the downregulation of *IL-8* and *NFƙB* genes in treated human colon cancer cells. Suppression of *survivin* and pro-inflammatory genes are significantly associated with the apoptosis-inducing and cell-growth-inhibiting activities of anticancer agents [18,34,35]. Considering several benefits of cell-free extracts of probiotic microorganisms and strong anticancer and antiproliferative characteristics of SBS against AGS cell lines, implementing in vivo and animal study models is suggested to explore the potential of SBS as a practical therapeutic strategy against stomach cancer in humans. Also, regarding the relationship between the expression of *survivin* gene, chromosome segregation, and cytokinesis in treated cells [36], evaluation of these effects are suggested to be implemented in future studies.

## 4. Materials and Methods

### 4.1. Saccharomyces Cerevisiae var. Boulardii Supernatant (SBS) Preparation

Lyophilized probiotic *S. boulardii* strain CNCM I-745 (Yomogi^®^, Mutaflor Co., Sydney, Australia) was ordered and purchased from a local pharmacy. According to the method we used previously for the preparation of SBS [5,6], 100 mg of lyophilized *S. boulardii* was dissolved, activated, and grown in 100 mL of yeast peptone dextrose broth (YPD, Sigma-Aldrich, Darmstadt, Germany) and incubated overnight at 37 °C. The incubated suspension was centrifuged for 15 min at 7400 rpm. The supernatant was collected and passed through a sterilized 0.2 µm filter (Sigma-Aldrich, MilliporeSigma Co., Darmstadt, Germany). The filtered supernatant was lyophilized and diluted with RPMI 1640 supplemented with FBS and antibiotics. SBS treatments were prepared in 200, 400, 800, 1600, and 3200 µg/mL concentrations (lyophilized dried SBS in standard RPMI 1640 cell medium culture).

### 4.2. Cell Culture

The human adenocarcinoma stomach cancer cell (AGS cell line) was purchased from the National Cell Bank of Pasteur Institute of Iran (Pasteur In., Tehran, Iran). The AGS cell line was activated in RPMI 1640 supplemented with FBS and antibiotics (the same concentrations as previously mentioned for SBS preparation) and incubated for 5 days at 37 °C with 5% CO_2_. The stock cell culture was prepared for subsequent experiments. Subcultures were transferred from the cells culture stock into the 96-well microplates and incubated for 3 days at 37 °C with 5% CO_2_ until reaching 80% confluence and the formation of cell monolayers. The cells were treated with different concentrations of SBS (each well contained 100 µL of standard RPMI 1640 cell medium culture and 100 µL of SBS treatment) and the same volume of dimethyl sulfoxide (each well contained 100 µL DMSO, this compound does not induce any significant cellular anti-proliferative effect, and 100 µL of standard RPMI 1640 cell medium culture as the negative control sample). After 24 and 48 h, treated cells were harvested for viability, mechanical properties, and gene expression analysis.

### 4.3. Cell Viability

The viability of stomach cancer cells treated with SBS was evaluated by using the MTT assay (3-(4,5-dimethylthiazol-2-yl)-2,5 diphenyl tetrazolium bromide) in this study. Medium culture in microplates was replaced with RPMI 1640 containing 0.5 mg/mL MTT and incubated for 4 h at 37 °C with 5% CO_2_. Then, DMSO was replaced with the medium culture containing MTT in each well. The level of color changes from yellow to purple due to the reduction of MTT to formazan in viable cells was measured at the absorbance of 570 nm in each well by using a microplate reader device model Elx808 (BioTek, Winooski, VT, USA). The cell viability percentage was calculated by using the following formula [18]:

Cell viability (%) = (E − N/C − N) × 100

where E, C, and N were the measured absorbance of the experiment, control, and blank samples, respectively. The IC_50_ value (inhibitory concentrations of 50%) was measured in the concentration of SBS treatment, which decreased the viability of stomach cancer cells to 50%. SBS IC_50_ concentration was considered to treat the cancer cells for mechanical properties and gene expression analysis.

### 4.4. Atomic Force Microscopy Analysis

In this study, atomic force microscopy (AFM) was used to investigate the nano-mechanical properties and morphological changes of the treated stomach cancer cells, as previously described by Zolghadr et al. [37]. Before AFM analysis, stomach cancer cells (106 cells) were seeded into the six-well microplates and incubated overnight at 37 °C with 5% CO_2_. The cells were exposed to SBS with the concentration of IC_50_ value and DMSO (as the control sample) and incubated for 24 and 48 h at 37 °C with 5% CO_2_. After incubation, the treated cells were washed two times with phosphate-buffered saline (PBS), and the cell fixation process was conducted by using glutaraldehyde (0.5% *w*/*v*) for 1 min. The glutaraldehyde solution was removed, and the cells were washed three times with PBS. After removing the PBS, the cells were dried at room temperature. A Hitachi AFM, model 5100N (MIKROMASCH-NSC15/AIBS, Tallinn, Estonia) with a V-shaped tip (side angel = 10°, radius = 10 nm, nominal spring constant = 0.07 − 0.35 N/m) and high sensitivity attached to the cantilever was employed in this study. The morphological properties of the cells were evaluated in non-contact mode at the temperature of 37 ± 1 °C. Standardization protocols, Young’s modulus calculation, image taking, and AFM data analysis were carried out as previously described by Zolghadr et al. [37].

### 4.5. Expression of Survivin and Pro-Inflammatory Genes

Expression of *survivin* and pro-inflammatory genes including nuclear factor ‘kappa-light-chain-enhancer’ of activated B-cells (*NFƙB*) and interleukin-8 (*IL-8*) were measured to evaluate the antitumor activity of SBS against human stomach cancer cells in this study. Reverse transcriptase real-time PCR and 2^-ΔΔCt^ assays were used to assess the expression levels of *survivin*, *NFƙB*, and *IL-8* genes in AGS cell lines treated with SBS after 24 and 48 h. Commercial CinaClon tissue RNA extraction kit (CinnaGen, Tehran, Iran) and commercial GeneAll cDNA synthesis kit (GeneAll Biotechnology Co., Seoul, Korea) accompanied with ABI PCR thermal-cycler machine model 9092 (Applied Biosystems, Bedford, MA, USA) were employed for total RNA extraction of treated samples and cDNA synthesis, respectively, according to the manufacturers’ instructions. *Survivin*, *NFƙB*, and *IL-8* primers were ordered and synthesized by SinaColon Company (SinaColon Co., Tehran, Iran). RotorGene real-time PCR machine model 6000 (Qiagen, Maryland, USA) and commercial Ampliqon real-time PCR SYBR green kit (Ampliqon, Odense, Denmark) were used for the real-time PCR method. The total real-time PCR reaction volume was 20 µL containing 10 µL of real-time PCR kit, 1 µL of each primer (20 µm/µL), 2 µL of cDNA template (50 ng/µL), and sterilized nuclease-free water up to the final reaction volume. Real-time PCR thermal cycling programs were performed as we previously described. The 2^−ΔΔCt^ assay was used to measure and calculate the relative gene expression levels [6,18].

### 4.6. Statistical Analysis

Analysis of variance (ANOVA) was used to measure the significant (*p* < 0.05) levels of difference between the variables by using SPSS version 23.0.0 (SPSS Inc., Chicago, IL, USA). All experiments and measurements were carried out in triplicates.

## 5. Conclusions

In conclusion, we demonstrate that SBS has cytotoxic and growth-inhibitory effects on the AGS cell line. SBS treatment also contributes to morphological changes in the cytoplasmic membrane of AGS cells. Expression of *survivin* and pro-inflammatory genes, including *IL-8* and *NFƙB*, are suppressed in AGS cells treated with SBS. Regarding the anti-proliferative and antitumor activities of SBS against the AGS cell line, it can be considered as a potential therapeutic strategy to treat human stomach cancer disease. However, the cell toxicity of SBS against different human intestinal normal cell lines is highly recommended to be evaluated in future studies.

## Figures and Tables

**Table 1:** AFM data analysis including Young`s modulus, adhesion force, and Z pulling values of treated and control AGS cells.

Treatment	Mean Young’s Modulus Value (kpa) ± SD *	Mean Adhesion Force (pN) ± SD	Mean Z Pulling (µm) ± SD
Control	0.95 ± 0.025 ^a^	148 ± 4.02 ^a^	0.61 ± 0.012 ^a^
After 24 h	1.57 ± 0.180 ^b^	128 ± 5.20 ^b^	1.23 ± 0.120 ^b^
After 48 h	1.62 ± 0.220 ^b^	112 ± 4.20 ^b^	1.58 ± 0.025 ^b^

* SD: standard deviation. Alphabetical letters in each column indicate significant differences (*p* < 0.05).

## Data Availability

We confirm that all data included in this research are available within the article.
